# Serum Levels of Human MIC-1/GDF15 Vary in a Diurnal Pattern, Do Not Display a Profile Suggestive of a Satiety Factor and Are Related to BMI

**DOI:** 10.1371/journal.pone.0133362

**Published:** 2015-07-24

**Authors:** Vicky Wang-Wei Tsai, Laurence Macia, Christine Feinle-Bisset, Rakesh Manandhar, Arne Astrup, Anne Raben, Janne Kunchel Lorenzen, Peter T. Schmidt, Fredrik Wiklund, Nancy L. Pedersen, Lesley Campbell, Adamandia Kriketos, Aimin Xu, Zhou Pengcheng, Weiping Jia, Paul M G. Curmi, Christopher N. Angstmann, Ka Ki Michelle Lee-Ng, Hong Ping Zhang, Christopher P. Marquis, Yasmin Husaini, Christoph Beglinger, Shu Lin, Herbert Herzog, David A. Brown, Amanda Sainsbury, Samuel N. Breit

**Affiliations:** 1 St Vincent’s Centre for Applied Medical Research, St Vincent’s Hospital and University of New South Wales, Sydney, NSW, Australia; 2 Centre for Immunology and Inflammation, School of Biomedical Sciences, Monash University, Clayton, VIC, Australia; 3 Discipline of Medicine University of Adelaide, Adelaide, SA, Australia; 4 Department of Nutrition, Exercise and Sports, Faculty of Science. University of Copenhagen, Frederiksberg C, Copenhagen, Denmark; 5 Department of Medicine, Unit of Gastroenterology and Hepatology, Karolinska University Hospital, Stockholm, Sweden; 6 Department of Medical Epidemiology and Biostatistics, Karolinska Institute, Stockholm, Sweden; 7 Diabetes and Metabolism Division, Garvan Institute of Medical Research, Sydney, NSW, Australia; 8 Department of Medicine & Department of Pharmacology & Pharmacy, University of Hong Kong, Hong Kong, China; 9 Department of Endocrinology and Metabolism, Shanghai Jiao Tong University Affiliated Sixth People’s Hospital, Shanghai Diabetes Institute, Shanghai Clinical Center of Diabetes, Shanghai, China; 10 School of Physics, University of New South Wales, Sydney, NSW, Australia; 11 School of Mathematics and Statistics, University of New South Wales, Sydney, NSW, Australia; 12 School of Biotechnology and Biomolecular Sciences, University of New South Wales, Sydney, NSW, Australia; 13 Clinical Research Center, Department of Biomedicine, University Hospital Basel, Basel, Switzerland; 14 Neuroscience Division, Garvan Institute of Medical Research, Sydney, NSW, Australia; 15 The Boden Institute of Obesity, Nutrition, Exercise & Eating Disorders, Sydney Medical School, The University of Sydney, Sydney, NSW, Australia; University of Alabama at Birmingham, UNITED STATES

## Abstract

The TGF-b superfamily cytokine MIC-1/GDF15 circulates in the blood of healthy humans. Its levels rise substantially in cancer and other diseases and this may sometimes lead to development of an anorexia/cachexia syndrome. This is mediated by a direct action of MIC-1/GDF15 on feeding centres in the hypothalamus and brainstem. More recent studies in germline gene deleted mice also suggest that this cytokine may play a role in physiological regulation of energy homeostasis. To further characterize the role of MIC-1/GDF15 in physiological regulation of energy homeostasis in man, we have examined diurnal and food associated variation in serum levels and whether variation in circulating levels relate to BMI in human monozygotic twin pairs. We found that the within twin pair differences in serum MIC-1/GDF15 levels were significantly correlated with within twin pair differences in BMI, suggesting a role for MIC-1/GDF15 in the regulation of energy balance in man. MIC-1/GDF15 serum levels altered slightly in response to a meal, but comparison with variation its serum levels over a 24hour period suggested that these changes are likely to be due to bimodal diurnal variation which can alter serum MIC-1/GDF15 levels by about plus or minus 10% from the mesor. The lack of a rapid and substantial postprandial increase in MIC-1/GDF15 serum levels suggests that MIC1/GDF15 is unlikely to act as a satiety factor. Taken together, our findings suggest that MIC-1/GDF15 may be a physiological regulator of energy homeostasis in man, most probably due to actions on long-term regulation of energy homeostasis.

## Introduction

The regulation of food intake, energy storage and energy expenditure is tightly controlled by complex homeostatic mechanisms. Total body nutritional and energy status, as well as the nutritional status in the gut lumen and circulation, is conveyed to the central nervous system (CNS), in large part via the release of hormones from the gut, pancreas and adipose tissue. The CNS, in turn, initiates the transcription and release of neuropeptides in the hypothalamus and brainstem, which then modulate feeding and metabolism to maintain energy homeostasis [1–3]. Gut- and adipose tissue-derived hormones may increase or decrease appetite. Satiety inducing gut hormones include cholecystokinin (CCK), amylin, glucagon-like peptide-1 (GLP-1), pancreatic polypeptide (PP) and peptide YY (PYY), which are released in response to food intake and act over short time frames [4]. Longer term appetite regulation involves mediators such as insulin from the pancreas, and leptin from fat [5]. In disease states, other regulatory molecules have also been identified as regulating energy homeostasis, raising the possibility that some of them may also be involved in physiological regulation of energy homeostasis. One such molecule is macrophage inhibitory cytokine/growth differentiation factor 15 (MIC-1/GDF15) [6], an unusual and divergent member of the TGF-b superfamily, also known as GDF15, PLAB, NAG-1 or PTGFB [6–8]. We have previously demonstrated that elevated circulating levels of this cytokine in disease states are an important cause of the anorexia/cachexia of cancer and other diseases [9].

MIC-1/GDF15 is a stress response cytokine that plays a role in several biological processes including cancer, chronic inflammatory diseases and energy homeostasis (reviewed in [10, 11]). The major source of circulating MIC-1/GDF15 is uncertain, but is probably the liver, although it is also expressed in other tissues including kidney, lung and adipose tissue. To exert a biological effect, MIC-1/GDF15 must activate its cognate receptor complex, which to this point has not been definitively identified. However, its receptor chains are likely to belong to the highly conserved heterodimeric TGF-b receptor (TBR) superfamily. There is some evidence for involvement of TBRII [12, 13], but as yet there is no genetic or direct biochemical data to confirm this and for this reasons its downstream signaling has not yet been clearly elucidated. Many different types of cancers express MIC-1/GDF15 causing elevation in its circulating levels [14–17]. In advanced cancers, serum MIC-1/GDF15 levels can rise dramatically, from the normal mean of about 450 pg/mL to 10,000–100,000 pg/mL [8, 14]. Elevated serum MIC-1/GDF15 levels have been linked to anorexia/cachexia associated with cancer [9], chronic renal [9] and cardiac failure [18, 19].

In experimental animals, MIC-1/GDF15 levels of above 5,000–8,000 pg/mL cause severe anorexia/cachexia [9] because of actions on feeding centres in the brainstem and hypothalamus [9, 20]. However, MIC-1/GDF15 is normally present in the blood of normal adult humans, with a broad normal range of 150 to 1150 pg/mL [16], which may rise substantially further in the elderly [21, 22]. This raises questions as to whether MIC-1/GDF15 levels within this normal circulating range may also regulate energy homeostasis. In support of this, our previous data demonstrated that MIC-1/GDF15 germline knockout mice (MIC-1^-/-^) have significantly increased visceral abdominal fat mass and weighed more than their syngeneic controls with a more pronounced obese phenotype in female than in male mice. Female MIC-1^-/-^ mice also ate significantly more and exhibited reduced basal energy expenditure. Moreover, when MIC-1^-/-^ mice were infused with sufficient recombinant MIC-1/GDF15 to raise their serum levels to the middle of the normal human range, their body weight and food intake were significantly reduced. These findings suggested that, at least in mice, physiological concentrations of MIC-1/GDF15 play a role in the regulation of appetite, fat deposition and body weight [23, 24].

To investigate whether MIC-1/GDF15 may also play a role in the regulation of energy homeostasis in humans, this study examined the relationship between body mass index (BMI) and serum MIC-1/GDF15 levels in a cohort of elderly identical twins. Additionally, circulating levels of MIC-1/GDF15 after ingestion of a high-energy meal were compared with the diurnal change in serum MIC-1/GDF15 levels. Lastly, interactions between MIC-1/GDF15 and several currently known gut-derived satiety factors were examined in healthy men.

## Materials and Methods

### Quantification of serum MIC-1/GDF15 levels

Collected serum or plasma samples were assayed for human MIC-1/GDF15 using a previously described, in house ELISA [25, 26].

### Ethics statement

All study participants provided written informed consent at the time of study enrollment and was approved by the relevant ethics committees.

#### Human twin studies

The ethics committees at Karolinska Institute and Umeå University approved the studies [22].

#### Circadian serum levels of MIC-1/GDF15

Institutional ethic committee of Shanghai Jiao Tong University approved the study protocol [27].

#### Fasting-refeeding studies in men

Sample collection was carried out at the Department of Human Nutrition, Faculty of Life Sciences, University of Copenhagen, Frederiksberg, Denmark, and was approved by the Municipal Ethical Commmittee of Copenhagen and Frederikberg in accordance with Helsiniki-II declaration (KF01-144/02) [28].

#### CCK and GLP infusion studies in men

The Royal Adelaide Hospital Ethic Committee approved the study protocol [29].

#### PYY infusion studies in men

The Ethics and Radiation protection committees of the Karolinska University Hospital, Sweden, approved the study [30].

### Human twin studies

The twin cohort has been previously described in several studies [22, 31, 32]. For the purpose of this study, the analysis was performed with serum from a group of 72 monozygotic non-obese twin-pairs. These were identified from the Swedish Twin Registry and had accessible serum samples, BMI data and self-reported life-style habits. Zygosity had been previously confirmed by either restriction fragment length polymorphism or serological testing. The twin pairs were aged between 63–93 years of age and had a BMI less than 30 kg/m^2^, and 69% of the pairs were female.

### Diurnal serum levels of MIC-1/GDF15

The diurnal pattern of serum MIC-1/GDF15 levels was determined in a cohort of 14 healthy men (*n* = 6) and women (*n* = 8) of Asian background and aged between 20 and 40 years, with a BMI ranging from 18 to 25 kg/m^2^. The serum samples from this cohort have been used in another study [27]. Briefly, all the subjects were housed in a ward of the Shanghai Sixth Hospital where they were allowed to freely walk around the ward, but not allowed to undertake strenuous activities. The lights were switched off during their sleep period of about 11:00 pm to 7:00 am. Subjects were provided with a standardized dinner at 1700 hours and were then fasted for 24 hours with free access only to water, from 1800 hours until 1800 hours on the following day. Blood samples were taken every 30 minutes during the period of fasting, from 1800 on day 1 to 1800 on day 2.

### Fasting-refeeding studies in men

Changes in serum MIC-1/GDF15 levels after fasting and following meal ingestion were determined in a cohort of 17 healthy male subjects aged between 18 and 50 years, with a BMI ranging from 24 to 31 kg/m^2^. The serum samples from this cohort have been used in another study and the experimental design has been previously described [28]. The study was approved by the Municipal Ethical Committee of Copenhagen and Frederiksberg, Denmark. Briefly, on the evening before commencement of the experiment, subjects were given a standardized meal to consume at no later than 2000 hours. They were not allowed to eat after that time, but were instructed to drink 0.25 L of water both before bed and in the morning before leaving their home. The subjects were instructed to avoid any heavy strenuous activities and alcohol 48 h before each test day. On the day of the experiment, subjects were provided with one of five isocaloric test meals, and these 5 meals were tested in these 17 subjects on 5 separate days in a randomized order. All test meals contained the same macronutrient composition with same portion of energy from protein (15%, casein powder + whey powder), fat (39%) and carbohydrate (46%), the differences between the meals are significant variations in mineral content: either high, medium or low amounts of calcium. The fourth meal contained low amount of calcium from dairy products and a calcium supplement served as a drink containing calcium carbonate. The fifth test meal contained soy protein powder as the only source of dairy protein and a low amount of calcium from dairy product. Each subject received portions that were matched to their individual energy requirements, which were determined using a formula based on a meta-analysis of 24-h calorimetry studies [28]. Each test meal contained 50% of the subject’s daily energy requirements. Blood samples were collected at 0, 30, 60, 90, 120, 180, 300 and 420 minutes after food intake.

### CCK and GLP infusion studies in men

The cohort used for this study has been previously described [29]. Briefly, this comprised 9 healthy male subjects aged between 18–27 years with a BMI between 20–25.2 kg/m^2^. After fasting overnight the subjects received intravenous infusions of isotonic saline, CCK-8 (1.8 pmol/kg/min) or GLP-1 (0.9 pmol/kg/min) or CCK plus GLP-1 at the same doses, in randomized, double-blind fashion on four different days separated by at least 3–10 days. The duration of the infusions was 150 minutes, and at *t* = 120 minutes, subjects were offered a standardized, cold buffet-style meal [29]. Subjects were allowed 30 min to consume the meal until they felt comfortably full. Blood samples were collected at 10-minute intervals for the first 30 minutes, at 15-minute intervals for the next 30 minutes, and at 30-minute intervals for the remaining 1200 minutes.

### PYY infusion studies in men

This cohort has been previously described [30]. Briefly, it comprised 8 healthy subjects (4 men, 4 women; age 28 ± 4 years; BMI 22 ± 2 kg/m^2^). Subjects were fasted overnight followed by ingestion of a 310 kcal egg omelet and a 70 kcal soft drink. This comprised energy from fat (57%), protein (22%), and carbohydrate (21%). On three different and separate study occasions, subjects received infusion of either 0.8 pmol/kg/min synthetic human PYY1-36 or PYY3-36 in 0.9% saline containing 0.1% albumin, or control (0.9 saline containing 0.1% albumin). Infusions started simultaneously with the meal intake and continued for 180 minutes. Blood samples were collected at 10-minute intervals for the first 60 minutes, followed by samples collected at 120 and 180 minutes.

### Statistics

All analyses were performed using GraphPad Prism (PRISM 4, GraphPad, San Diego, CA). In the twin cohort study, within-pair differences in BMI and MIC-1/GDF15 serum level were determined. These differences were plotted against each other and the correlation was analysed using Spearman regression, as data were not parametric.

For experiments studying postprandial serum levels of MIC-1/GDF15, measurements were obtained from 17 subjects that had 5 different meals on 5 separate occasions. The averages of MIC-1/GDF15 serum level from all subjects between 5 meals were compared by two-way repeated measure ANOVA. The average of the serum MIC-1/GDF15 level from the 5 meals were combined and then further analysed with a one-way ANOVA for time dependent interaction. Data from cohorts infused with CCK, GLP, or PYY were analysed by two-way repeated measures ANOVA with Bonferroni correction for multiple comparisons. Results are presented as means ± s.e.m. For all statistical tests, *p* values < 0.05 were considered to be statistically significant.

The diurnal rhythm of serum MIC-1/GDF15 levels for the i^th^ subject was modeled by the equation

$$L_{i}\left(t\right)+\varepsilon_{i}\left(t\right)=a_{i}\text{cos}\left(\frac{2\pi }{24}t+\phi_{i}\right)+b_{i}$$

Here *L*_*i*_ *(t)* is the level of MIC at time *t*, *α*_*i*_, *ϕ*_*i*_ and *b*_*i*_ are constants, and *ε*_*i*_ *(t)*, the error in the measurement, is a zero mean Gaussian noise. Each individual time series is fit to this function. The fits were performed using “NonlinearModelFit” in Mathematica. The model assumed Normally distributed error terms, so the residuals were tested to see if they were Normal. To a significance of 0.05, the residuals from all but subjects 11, 12, and 14 were Normally distributed. In the latter subjects, the presence of a small number of large outliers was responsible for this finding.

To test whether acrophase times are clustered, we performed Kuiper’s test [33] against the hypothesis that the maxima occur at random times between 0 and 24 hours. The resulting test statistic was 0.506, which gives a p-value of 0.00094, hence the null hypothesis that the acrophase times are distributed according to a uniform distribution is rejected at the 0.05 level.

## Results

### Human MIC-1/GDF15 serum levels are inversely correlated with BMI

**Fig 1:**
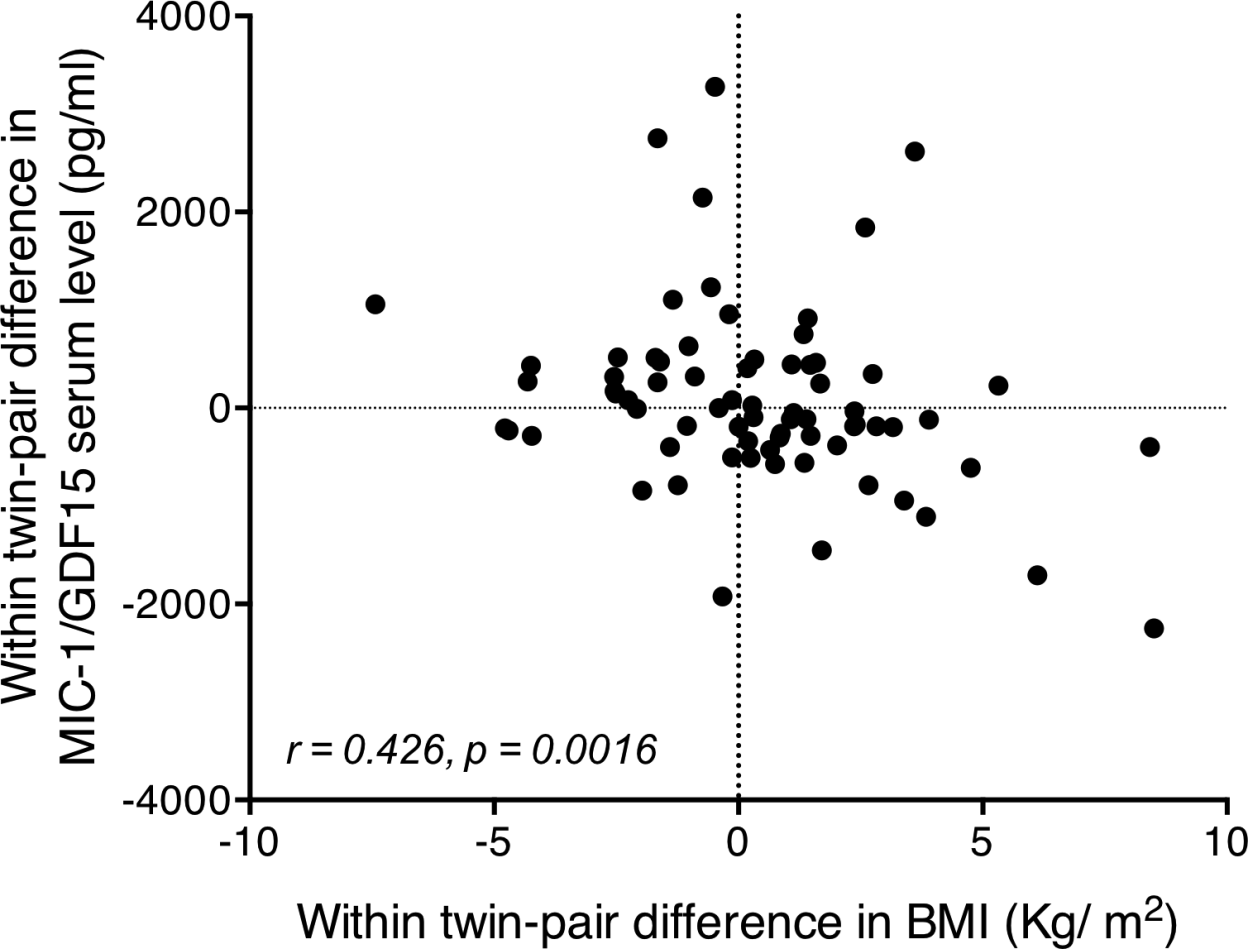
Correlation of monozygotic within-pair differences in MIC-1/GDF15 serum levels and within-pair differences in BMI. Correlation between within twin-pair difference in serum MIC-1/GDF15 levels and within twin-pair difference in BMI (*n* = 72 twins), performed by Spearman regression, identifies a highly significant correlation. In general the twin of the twin pair with a higher serum MIC-1/GDF15 level generally had a lower BMI than their identical twin pair. The reverse was also true.

To investigate whether MIC-1/GDF15 may regulate energy homeostasis in humans, as in mice, we examined the relationship between MIC-1/GDF15 serum levels and BMI. To increase the sensitivity of our analysis we examined genetically identical, non-obese monozygous twins from the Swedish twin cohort [21, 22, 31] with the aim of eliminating the effect of heritable variables and reducing disease-related confounding factors. The within-pair difference in serum MIC-1/GFDF15 levels was inversely correlated with the within-pair difference in BMI (*n* = 72 twin pairs, *r = 0*.*426 and p* = 0.0016) (Fig 1). For example, this means that when one twin had a higher serum MIC-1/GDF15 level than his/her identical sibling, the BMI was generally lower than that of the sibling. The reverse was also true. These data suggest that in non-obese human subjects, MIC-1/GDF15 may regulate BMI independently of genetic background. Furthermore, the observed correlation between MIC-1/GDF15 serum levels and BMI in humans suggests that the data we have previously obtained in mice, demonstrating that high MIC-1/GDF15 levels lead to weight loss and that absent MIC-1/GDF15 levels leads to weight gain[23], may also apply to humans.

### Human MIC-1/GDF15 serum levels are modulated in a diurnal pattern

**Fig 2:**
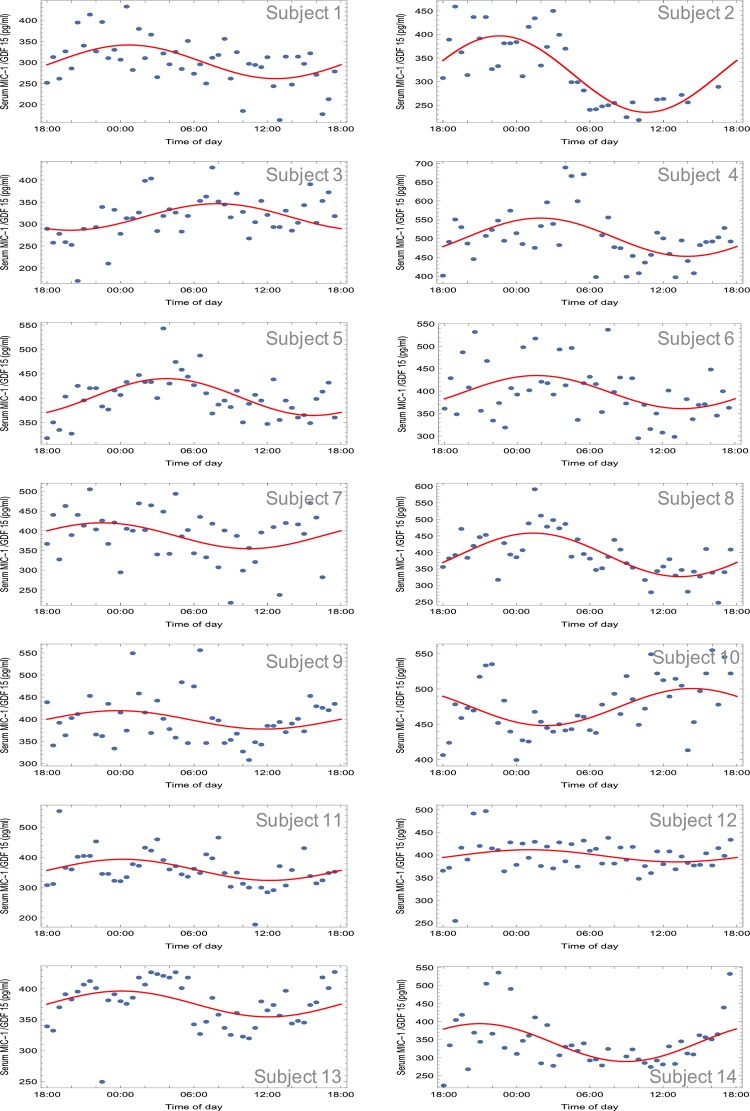
Fasting diurnal variation of human MIC-1/GDF15 serum levels. Serum MIC-1/GDF15 levels were measured on blood sample taken from participants every 30min during a 24h period. The oscillatory pattern of serum MIC-1/GDF15 levels for each individual subject was fitted to a cosine curve function.

To determine whether circulating MIC-1/GDF15 is subject to diurnal variation, we studied an Asian cohort from whom blood was drawn periodically over a single 24-hour cycle [27] and analyzed for serum MIC-1/GDF15 (Fig 2 and Table 1). These data indicate a biphasic pattern of serum MIC-1/GDF15 over 24 hours, with a mean serum level of 387.0 ± 4.2 pg/mL. For 12 of the 14 subjects, circulating MIC-1/GDF15 levels showed a diurnal variation with a peak around midnight and a nadir approximately at noon. The remaining two subjects (subjects 3 and 10, Fig 2 and Table 1) also show a diurnal variation, however, these cycles are out of phase with the majority (by 8 and 14 hours, respectively). For each subject, the diurnal variation in serum MIC-1/GDF15 serum levels could be fitted to a cosine curve (see Methods) and the acrophase times were clustered rather than occurring at random times over the 24 hour cycle (Kuiper’s test 0.506; p<0.0001). For the 12 subjects that are approximately in phase, the acrophase (clock time of maximal value) occurred at 0022 h (standard deviation of 1 hr 48 minutes) and the nadir at 1222 h (standard deviation of 1 hr 48 minutes). For these subjects, circulating MIC-1/GDF15 levels reach a peak of 422 ± 15 pg/mL at acrophase, and a minimum value of 341 ± 17 pg/mL at the nadir (Fig 2 and Table 1). The 24-hour cosine amplitude (half of the peak-nadir difference) was 381 ± 41 pg/mL, and the MESOR (midline estimating statistic of rhythm) was 381 ± 15 pg/mL. Thus, this diurnal oscillation leads to a variation in circulating MIC-1/GDF15 concentration about the MESOR of about ± 10%.

**Table 1:** Time of day for maximum and minimum MIC-1/GDF15 serum levels.

Subject	Time of Maximum	Time of Minimum
1	00:40	12:40
2	22:36	10:36
3	07:53	19:53
4	01:57	13:57
5	03:44	15:44
6	01:36	13:36
7	22:29	11:45
8	01:22	13:22
9	23:45	11:45
10	14:21	02:21
11	00:11	12:11
12	01:03	13:03
13	00:04	12:04
14	20:57	08:57

### Postprandial changes of human MIC-1/GDF15 serum levels are not significantly different from its diurnal variation

**Fig 3:**
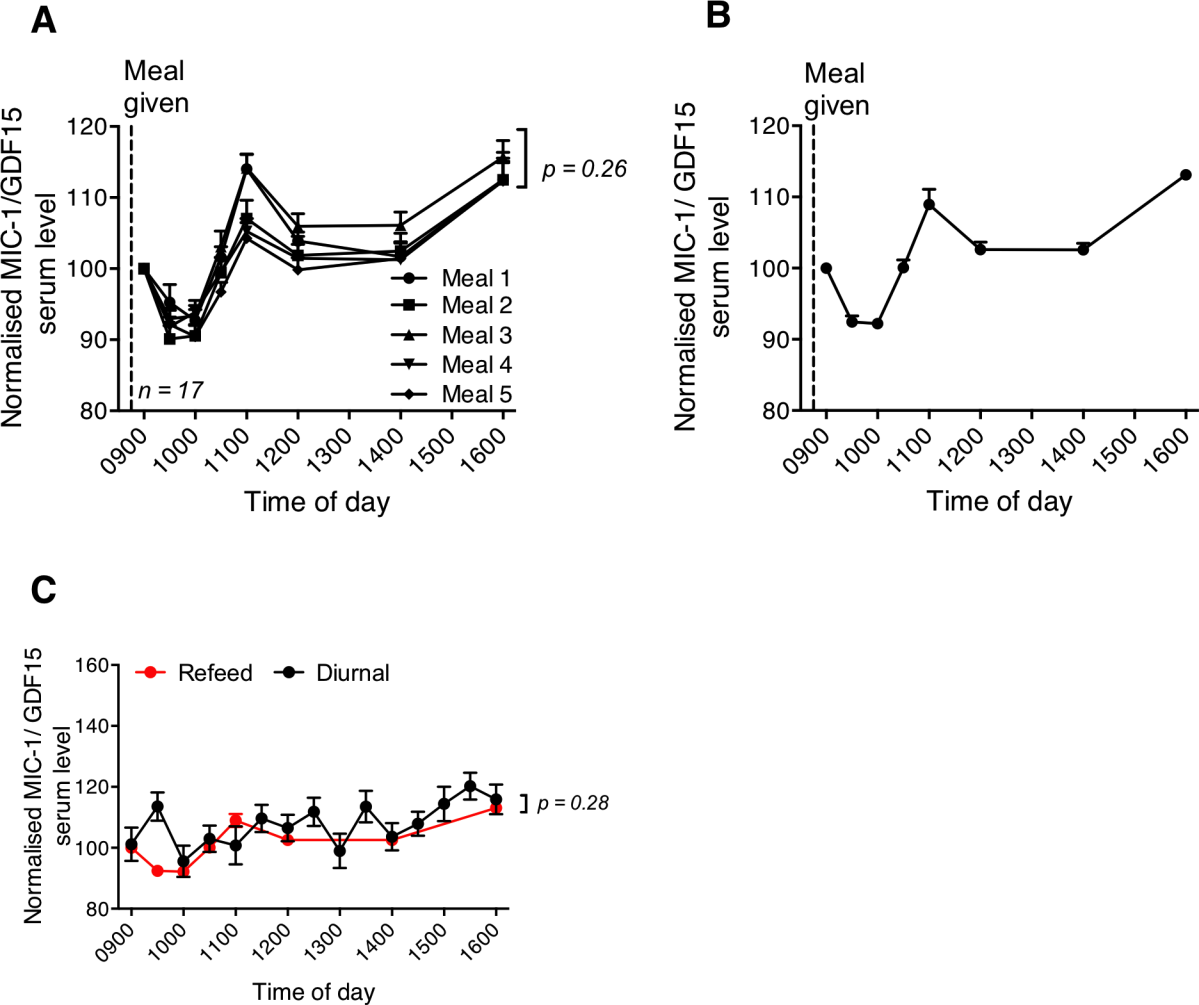
Postprandial changes in human MIC-1/GDF15 serum levels. Changes in serum MIC-1/GDF15 levels over time were measured in 17 subjects that received 5 different isocaloric meals on 5 separate occasions. (A) Changes in MIC-1/GDF15 serum levels do not differ for the 5 subjects fed the 5 different meals (*n* = 17, *p* = 0.26 *repeated measure ANOVA*). (B) When average data from all meals was pooled and normalised to baseline concentrations, there was significant time dependent alteration in circulating MIC-1/GDF15 levels (*n* = 5 meal; 17 subject/meal, *p* < 0.001 *one-way ANOVA*). (C) The postprandial profile of MIC-1/GDF15 serum levels (red) described in panel B were not significantly from its 24 h oscillatory pattern (*p* = 0.28, *repeated measure ANOVA*). Data represented as mean ± s.e.m.

One mechanism by which MIC-1/GDF15 might influence body weight and food intake is by promoting satiety. If this were the case, food intake might lead to a substantial rise in serum MIC-1/GDF15 levels. To evaluate this, circulating MIC-1/GDF15 levels were measured in a fasting refeeding cohort in which samples were drawn between 0900 and 1600 h of day. The average response of the 17 subjects to all 5 different meals showed a similar pattern of postprandial serum MIC-1/GDF15 levels over time, which did not vary significantly between the 5 meals (Fig 3A; *p* = 0.26). On average for the 5 different meals (Fig 3B), MIC-1/GDF15 serum concentrations decreased to a minimum of 92.2 ± 0.7% of fasting levels at 30 to 60 minutes post meal (0 *vs* 30 minutes or 60 minutes, *p* < 0.01). It then rose to peak at 108.9 ± 2.1% of fasting levels at 120 minutes post meal (90 *vs* 120 minutes, *p* < 0.01), after which it dropped to 102.6 ± 1.1% at 180 and 300 minutes post meal, which was not significantly different from the fasting concentration (0 *versus* 180 min or 300 minutes, *p* > 0.06). MIC-1/GDF15 serum concentrations, then increased significantly to 113 ± 0.7% of fasting values at 420 minutes post meal (300 *versus* 420 minutes, *p* < 0.001).

These undulating changes in serum MIC-1/GDF15 concentrations in the 7 hours after a meal made us question whether the changes in concentrations were due to the meal itself, or due to an underlying diurnal variation. Indeed, comparison of serum MIC-1/GDF15 level between the postprandial fasting refeeding cohort with samples from the diurnal cohort, collected fasting (13–20 hours after feeding) and at the same time of the day (0900 to 1600 h; Fig 3C) were very similar. A gradual rise in serum MIC-1/GDF15 concentrations was observed from 1000 h in both groups of subjects, and these patterns were not significantly different from each other (meal * fasting: *p* = 0.28, *repeated measures ANOVA*). This result shows that although there are small changes in MIC-1/GDF15 concentration in the hours after food intake, these could at least in part be accounted for by the underlying diurnal variation pattern.

### Effect of CCK and GLP-1 infusion on serum levels of MIC-1/GDF15

**Fig 4:**
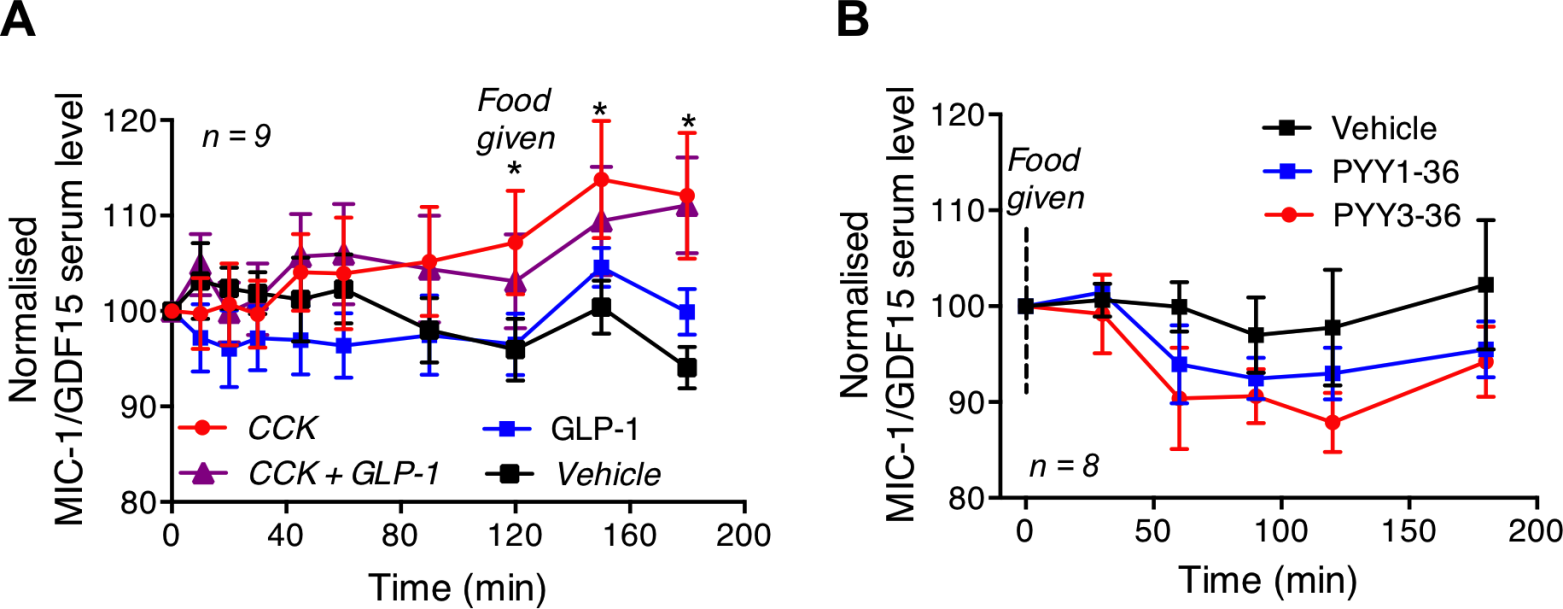
Effect of satiety factor infusions on serum levels of human MIC-1/GDF15. (A) Subjects receiving CCK-8 infusion alone had significant time dependent increase in serum levels of MIC-1/GDF15 with significant increases at 120, 150 and 180 minutes of infusion. Infusion of GLP-1 or CCK plus GLP-1 had no significant effect on serum MIC-1/GDF15 level (*n* = 9, *vehicle vs GLP-1*, *p* = 0.2; *vehicle vs CCK-8 + GLP-1*, *p* = 0.06). (B) PYY1-36 or PYY3-36 or saline was infused in subjects that were fasted overnight and had a 310-Kcal meal. Neither PYY1-36 nor PYY3-36 infusions had a significant effect on serum MIC-1/GDF15 levels (*n* = 8, *vehicle vs PYY1-36*, *p* = 0.18; *vehicle vs PYY3-36*, *p* = 0.34). Data were analysed by ANOVA *with Bonferroni correction* and are presented as mean ± s.e.m. * represents *p* < 0.05.

To evaluate whether circulating satiety hormones might modulate serum MIC-1/GDF15, we measured its levels in subjects who were infused intravenously with either vehicle, CCK-8, GLP-1, or CCK-8 plus GLP-1. For comparison purposes the serum MIC-1/GDF15 level for each participant was normalized to the value obtained at time zero (Fig 4). Compared to vehicle, subjects receiving CCK-8 infusion alone exhibited a small, but significant, time dependent increase in serum levels of MIC-1/GDF15 (treatment * time: *p* = 0.001, repeated measures ANOVA, Fig 4A). This change was particularly notable after consumption of the buffet meal, because a significant increase in circulating MIC-1/GDF15 levels was found at 120, 150 and 180 minutes after commencement of CCK-8 infusion (vehicle *versus* CCK, n = 8; 120 min; *p* = 0.05, 150 min; *p* = 0.01, 180 min; *p* < 0.001 repeated measure ANOVA with Bonferroni correction for multiple testing). MIC-1/GDF15 serum levels were not modulated by GLP-1 infusion alone (vehicle *versus* GLP-1, treatment *p* = 0.6; treatment * time, *p* = 0.2, repeated measures ANOVA), nor did CCK plus GLP-1 have any significant effect on serum levels of MIC-1/GDF15 (vehicle *versus* CCK-8 + GLP-1, treatment, *p* = 0.36; treatment * time, *p* = 0.06, repeated measure ANOVA). The CCK8 infusion associated changes in MIC-1/GDF15 serum levels are very small, not likely to be biologically significant and may be well accounted for by the underlying diurnal variation.

### Effect of PYY infusion on serum levels of MIC-1/GDF15

To ascertain whether the satiety factor PYY may modulate MIC-1/GDF15, we measured its serum levels in healthy subjects infused with vehicle, PYY1-36 or PYY3-36 on 3 separate occasions. For comparison purposes the serum MIC-1/GDF15 level for each person was normalized to the value obtained at time zero. Infusion of the full length PYY (PYY1-36) had no significant effect on serum MIC-1/GDF15 concentrations (n = 8, vehicle versus PYY1-36, treatment, *p* = 0.18; treatment * time, *p* = 0.18, repeated measures ANOVA) (Fig 4B). Similarly, infusion of the truncated PYY (PYY3-36) had no effect on the serum levels of MIC-1/GDF15 (vehicle versus PYY3-36, treatment, *p* = 0.34; treatment * time, *p* = 0.4, repeated measures ANOVA). These findings suggest that neither PYY1-36 nor PYY3-36 has a significant effect on serum levels of MIC-1/GDF15.

## Discussion

This study provides the first evidence that circulating concentrations of MIC-1/GDF15 vary in a diurnal pattern. Further, consistent with our animal data [23], there was a significant correlation between its serum concentrations and BMI in monozygotic twin pairs, suggesting that it may also have a physiological role in the regulation of energy homeostasis in humans. Since we could not identify any direct evidence for the substantial postprandial changes in MIC-1/GDF15 serum level, we suggest that MIC-1/GDF15 is unlikely to be a satiety factor and is most likely to regulate long term energy balance.

Our previous studies of MIC-1/GDF15’s role in energy homeostasis under physiological conditions, have been undertaken in mice. Studies in inbred mouse lines including transgenic and gene knockout mice allow us to control for the major changes that can occur because of genetic variations between individuals. In humans, the best way to control for genetic variations is to study monozygotic twins. In this study we have been able to capitalize on the genetic identity of monozygotic twins, but at the same time, utilize the small phenotypic differences in BMI that develop within non-obese twin pairs, to show that there is a significant relationship between the within-pair difference in serum levels of MIC-1/GDF15 and the within-pair difference in BMI such that the twin of the twin pair with the higher MIC-1/GDF15 serum level usually has the lower BMI and visa versa. One limitation in this sub study is a relative lack of data regarding health status in twins that might influence serum MIC-1/GDF15 levels. However, as we, and others, have shown, significant relationships with circulating MIC-1/GDF15 levels and BMI in a range of diseases [9, 14, 34–36], in this context, we do not believe it is a major limitation. Therefore, independent of heritable factors, the consistent data between humans and mice suggests that MIC-1/GDF15 has a conserved role in regulating body weight.

It is common for serum levels of cytokines, such as IL-6 and TNF-alpha, to rise with age and MIC-1/GDF15 are not an exception. Its serum levels are significantly higher in elderly cohorts like ours [22, 34] [37] and aging itself seems to be the major non-genetic contributing factor associated with elevated serum MIC-1/GDF15 [14, 37]. A previous study performed in the same monozygotic twin cohort [22] showed that unique rather than shared environmental factors might influence serum MIC-1/GDF15, and these environmental factors may be those associated with ageing such as oxidative stress or elevation of levels of other cytokines.

Our studies have also identified that in line with many metabolic modulators, serum levels of MIC-1/GDF15 vary in a bimodal diurnal pattern. In humans of Asian background, we found that the 24-hour profile of circulating MIC-1/GDF15 levels show a trough in concentration occurring at about 1222 h and a peak in concentration occurring at around 0022 h. Fasting and then refeeding at 0900 h does not significantly modify this pattern (Fig 3). However, it is possible that some of this diurnal variation is entrained to meal patterns, as has been noted for circulating levels of energy regulatory hormones, such as leptin [38]. Therefore, additional diurnal profiling of circulating MIC-1/GDF15 after prolonged fasting would be needed to determine if its diurnal profile is entrained to meal patterns or follows an independent circadian pattern. Interestingly, a similar diurnal pattern to that of MIC-1/GDF15 has been previously observed for leptin [39, 40], in which the highest circulating levels were reached after midnight, and the nadir occurred at around noon. However, the nocturnal rise in circulating leptin was totally eliminated in subjects fasted for 72 hours prior to sample collection [41], indicating that the rhythm in daily leptin concentrations was entrained by meals. The nocturnal rise in MIC-1/GDF15 seen in the current study also resembles the rise in growth hormone (GH) secretion and the increase in circulating free fatty acids (FFAs) that have been seen in other publications [40]. Interestingly, GH secretion is known to profoundly affect body composition, and as body composition is a phenotypic change in MIC-1^-/-^ mice [23], it is conceivable that there is a relationship between the secretion of MIC-1/GDF15, leptin and GH. This is further supported by an earlier study showing that mice bearing MIC-1/GDF15 overexpressing tumors have reduced hypothalamic expression of mRNA for growth hormone releasing hormone (GHRH) [9].

The area postrema (AP) and nucleus of the solitary tract (NTS) in the hindbrain are crucial regions for the anorexigenic actions of MIC-1/GDF15 [20]. These regions are also major sites of action for satiety factors such as CCK-8, GLP-1 and PYY. For example, the CCK-1 (or, A) receptor is expressed in these regions, as well as in the dorsal medial hypothalamus (DMV) and in the vagal afferent neurons of the stomach and small intestine [42, 43]. One of the major characteristics of these satiety factors is that their circulating levels are rapidly and substantially elevated post-prandially, due to food-induced secretion from the gastrointestinal tract. For example, both PYY and GLP-1 are expressed by the intestinal endocrine cells and are rapidly released into the circulation after a meal. Based on this parallel between the central sites of action of MIC-1/GDF15 and known satiety hormones, it seemed plausible that MIC-1/GDF15 might have satiety factor-like activity. Whilst we did not test directly for satiety factor activity of MIC-1/GDF15, we would expect to observe a rapid and substantial postprandial variation in its serum levels similar to that seen for other satiety factors. However, our studies indicated that circulating MIC-1/GDF15 levels were increased by only 10% from baseline values at two hours post re-feeding. This modulation was small and delayed, and thus inconsistent with what would be expected of a satiety factor. Further, these small changes in serum levels of MIC-1/GDF15 in response to the meal are at least in part accounted for by its underlying diurnal variation pattern. Based on this data MIC-1/GDF15 secretion into the blood does not have a pattern consistent with that of a satiety factor.

To investigate whether known satiety factors might modulate serum levels of MIC-1/GDF15, we examined serum from subjects that had been infused with CCK-8, GLP-1, PYY1-36 or PYY 3–36. Of these, only CCK-8 was associated with a significant alteration in MIC-1/GDF15 serum levels relative to vehicle infusion. Indeed, when CCK was infused to mimic the postprandial rise in its circulating levels, there was a 10% increase in serum levels of MIC-1/GDF15 from its baseline at 120 minutes after commencement of infusion. This coincided with the increase in serum MIC-1/GDF15 levels observed at 120 minutes after meal ingestion, suggesting that a postprandial increase in circulating CCK-8 levels could have mediated the small postprandial increase in circulating MIC-1/GDF15 levels. Thus, it is possible that the small MIC-1/GDF15 level changes in response to meals, may be mediated in part by CCK-8.

In summary, our study suggests that MIC-1/GDF15 may play a role in human physiological regulation of body weight, a finding that is consistent with data generated from mouse studies. Also consistent with this, serum levels of MIC-1/GDF15 vary in diurnal pattern, similar to that of leptin, a well-known regulator of long-term energy homeostasis. The absence of substantial postprandial changes of MIC-1/GDF15 serum levels, distinct from its underlying diurnal variation in concentrations, suggest that MIC-1/GDF15 is unlikely to have significant satiety factor like activity, but rather acts as a long-term regulator of energy homeostasis.
